# Impact of Nitroglycerin Administration on Acetylcholine Provocation Testing in Angina With Nonobstructive Coronary Arteries

**DOI:** 10.1016/j.jscai.2025.103668

**Published:** 2025-07-23

**Authors:** Rajan Rehan, Christopher C.Y. Wong, James Weaver, Pankaj Jain, Mark Adams, Martin K.C. Ng, Jennifer A. Tremmel, Andy S.C. Yong

**Affiliations:** a Department of Cardiology, Royal Prince Alfred Hospital, Sydney, New South Wales, Australia; b Department of Cardiology, Concord Hospital, Sydney, New South Wales, Australia; c Sydney Medical School, University of Sydney, New South Wales, Australia; d Division of Cardiovascular Medicine, Stanford University School of Medicine, Stanford, California; e Stanford Cardiovascular Institute, Stanford, California; f Faculty of Medicine, Health and Human Sciences, Macquarie University, New South Wales, Australia

**Keywords:** acetylcholine provocation testing, acetylcholine rechallenge, angina with nonobstructive coronary arteries, coronary artery spasm, coronary function testing

## Abstract

**Background:**

Invasive coronary function testing, using acetylcholine (ACh) to diagnose coronary artery spasm (CAS) and coronary microvascular dysfunction assessment, is considered the gold standard for evaluating patients suffering from angina with nonobstructive coronary arteries. Notably, equipoise remains regarding the optimal sequence for coronary function testing, and no global consensus exists. Although nitroglycerin (NTG) is routinely administered post–radial access and prior to coronary microvascular dysfunction testing, its effect on subsequent ACh testing remains unclear. This study aimed to evaluate the diagnostic impact of preceding intravascular NTG on ACh provocation testing.

**Methods:**

Multivessel ACh provocation testing was systematically performed in patients with suspected CAS. To assess the reinducibility of epicardial spasm, an ACh rechallenge was performed in patients who tested positive by readministering the spasm provocation dose into the affected coronary artery at different time intervals following the administration of intravascular NTG.

**Results:**

This multicenter study enrolled 102 patients (mean age 59.3 ± 10.0 years; 55% female), of whom 40 were diagnosed with epicardial CAS and underwent ACh rechallenge. Among these, 25 patients (62.5%) exhibited a diffuse spasm pattern, whereas 15 patients (37.5%) demonstrated focal spasm. After the ACh rechallenge, epicardial spasm was reinduced in 22 patients (55%), microvascular spasm in 6 patients (15%), and no spasm in 12 patients (30%). The sensitivity of ACh provocation testing declined to 55% at the end of the rechallenge.

**Conclusions:**

Nitroglycerin administration reduces the diagnostic accuracy of ACh provocation testing for CAS in angina with nonobstructive coronary arteries patients. Findings from this study indicate that clinicians should avoid NTG administration prior to ACh testing or significantly delay ACh testing after NTG exposure to preserve diagnostic sensitivity.

## Introduction

Nearly half of all patients undergoing coronary angiography for stable angina are found to have nonobstructive coronary arteries.^1^ Invasive coronary function testing (CFT), using acetylcholine (ACh) to diagnose coronary artery spasm (CAS) and a pressure-temperature sensing guide wire to evaluate for coronary microvascular dysfunction (CMD), is considered the gold standard for evaluating patients with angina with nonobstructive coronary arteries (ANOCA).^2^^,^^3^ These investigations carry a class Ib recommendation in the European Society of Cardiology guidelines, supporting a tailored therapeutic approach that has been shown to enhance angina control and improve quality of life.^4^^,^^5^

The optimal sequence for CFT remains a subject of debate, with many centers opting to perform ACh provocation testing as the initial step.^6^, ^7^, ^8^, ^9^ In this approach, nitroglycerin (NTG) is administered after CAS testing, followed by insertion of an intracoronary pressure wire and administration of adenosine for CMD assessment. Conversely, other clinicians advocate for CMD testing first, as they believe that ACh-induced spasm may alter baseline microvascular resistance and overall coronary flow, potentially compromising the accuracy of those results.^10^^,^^11^ This alternative approach requires NTG administration prior to ACh provocation, which can potentially lead to a false negative test for CAS.^12^^,^^13^ Furthermore, the widespread adoption of radial access in most catheterization laboratories, where intraradial NTG and/or calcium channel blockers are routinely administered to prevent radial artery spasm, may obscure accurate assessment of CAS. Although NTG is considered short-acting, its administration following radial access but prior to ACh testing may further contribute to the underdiagnosis of CAS.

Given these differing perspectives, the exact sequence of steps during CFT varies across centers and there is no consensus on the optimal protocol. This study aims to evaluate the diagnostic impact of preceding intravascular NTG on ACh provocation testing.

## Methods

This multicenter, prospective study assessed the effect of intravascular NTG on ACh provocation testing in ANOCA patients from August 2022 to June 2024 (https://anzctr.org.au/; Unique identifier: ACTRN12622001521718). All patients were referred by their treating cardiologist for suspected ANOCA. Diagnostic invasive coronary angiography was performed to confirm the absence of obstructive coronary artery disease in all patients, defined as a diameter stenosis of less than 50% by visual estimation. Patients underwent ACh provocation testing in the left (LCA) and right (RCA) coronary arteries followed by adenosine-mediated coronary physiology assessment (Supplemental Table S1) with a minimum interval of 10 minutes between both procedures. For safety reasons, ACh provocation testing was avoided in cases where the coronary arteries were nondominant, or severely tortuous. Patients were eligible for inclusion if they had evidence of epicardial CAS confirmed by ACh provocation testing. Exclusion criteria included recent (within 3 weeks before cardiac catheterization) acute coronary syndrome, prior heart transplantation, coronary artery bypass grafting, serum creatinine >1.5 mg/dL, and/or inability to provide informed consent.

Clinical data on patient characteristics, cardiac risk factors, and symptom profiles were collected prior to the procedure. Both traditional cardiovascular risk factors and nontraditional variables associated with vasomotor disorders were documented. The study protocol adhered to the ethical guidelines of the 1975 Declaration of Helsinki and received approval from the human research ethics review board. Written informed consent was obtained from all participants. R.R. and A.S.C.Y. had full access to all study data and were responsible for ensuring data integrity and conducting the analysis.

### ACh provocation testing protocol

All ACh provocation testing was conducted in the morning to ensure standardized patient timing. Patients were requested to withhold vasoactive medications (eg, calcium channel blockers and long-acting nitrates) and substances containing methylxanthine for >4 times the duration of the drug half-life. Continuous 12-lead electrocardiogram (ECG) monitoring was employed throughout the procedure. Diagnostic invasive coronary angiography was performed per standard institutional practice via the radial or femoral artery. Administration of intravascular vasodilator drugs (eg, NTG, calcium channel blockers) was avoided following initial arterial access prior to ACh provocation testing in all patients. After confirming the absence of obstructive coronary artery disease, multivessel ACh provocation testing was conducted.

To compensate for potential bradycardia, a temporary transvenous pacemaker was inserted via the femoral vein, activated only if a pause exceeding 5 seconds occurred. A 6F angioplasty guiding catheter without side holes was then positioned in either the LCA or RCA, guided by clinical judgment. For the LCA assessment, incremental doses of 20, 50, 100, and 200 μg of ACh were injected over 20 seconds, with a 2-minute gap between doses. After each injection, cine-images were obtained to assess changes in coronary diameter through quantitative coronary angiography offline using standard commercial software on a Leonardo workstation (Quant, Siemens), which is derived from the CAAS II system (Pie Medical Imaging).^14^ If CAS was induced with reproducible symptoms and ST-segment changes (see definitions below), the provocation test was terminated and concluded to be positive. For the RCA, a similar protocol was followed with incremental doses of 20, 50, and 80 μg of ACh. Intracoronary NTG was administered to reverse the effects of ACh following the completion of testing.

### ACh rechallenge protocol

**Figure 1:**
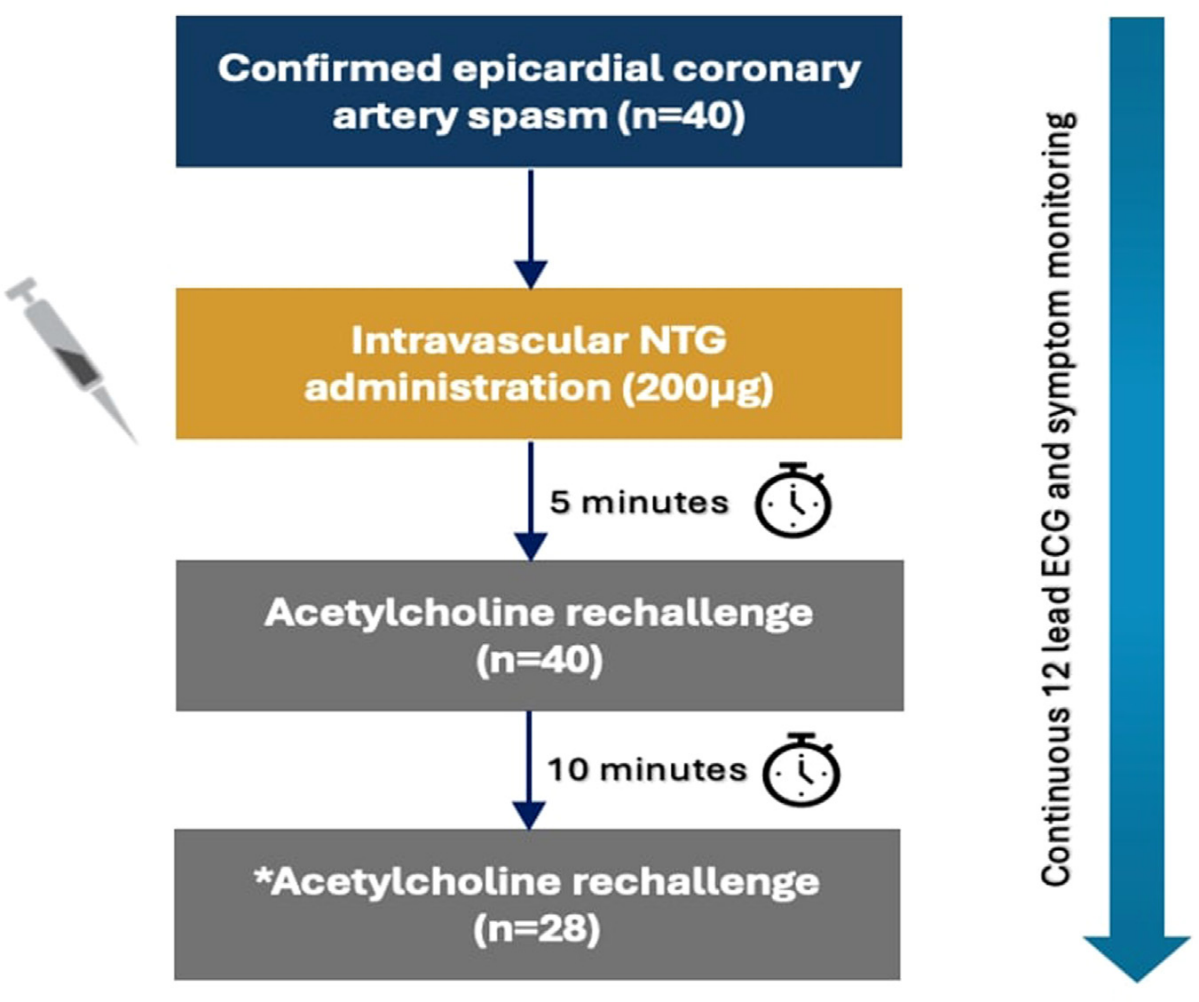
**Study flowchart.** Protocol for acetylcholine (ACh) rechallenge in patients with confirmed epicardial coronary artery spasm. ∗Only patients (n = 28) who did not demonstrate epicardial coronary artery spasm at the 5-minute ACh rechallenge proceeded to the 10-minute ACh rechallenge. ECG, electrocardiogram, NTG, nitroglycerin*.*

To assess the reinducibility of epicardial spasm, the ACh rechallenge was performed in patients who tested positive by readministering the spasm provocation dose into the affected coronary artery. Following the administration of intraradial NTG (200 μg), the ACh rechallenge was performed at 5 minutes, with a second challenge at 10 minutes if no epicardial spasm was detected during the initial assessment (Figure 1). Patient symptoms, ischemic ECG changes, and coronary artery diameter reduction were monitored similarly to the initial assessment. Patients with an indeterminate result during the ACh rechallenge, defined as symptom reproduction without either >90% vasoconstriction or ECG changes, were categorized as having no spasm.

### Definitions

Coronary artery spasm was defined as per previously published international consensus.^15^ Epicardial spasm was defined as a focal or diffuse epicardial coronary artery diameter reduction >90% in response to ACh compared with the relaxed state, with a reproduction of recognizable symptoms and/or ischemic ECG changes. Microvascular spasm was diagnosed when there was a reproduction of recognizable symptoms with ischemic ECG changes in the absence of >90% diameter reduction in response to ACh. Ischemic ECG changes were defined as transient ST-segment elevation or depression of >0.1 mV, or ischemic T-wave changes, in at least 2 contiguous leads.

### Statistical analysis

Continuous data are presented as mean ± SD, or median and IQR, as appropriate. Categorical data are presented as counts and percentages. The χ^2^ and Fisher exact tests were used to compare categorical variables. The *t* test and the Mann-Whitney *U* test were used to compare continuous variables as appropriate. A significance threshold of *P* < .05 was used to determine statistical significance. All analyses were performed using R version 4.2.2 (R Foundation for Statistical Computing).^16^

## Results

This study was conducted across 2 tertiary referral centers and included 102 patients, 40 of whom were diagnosed with epicardial CAS and underwent ACh rechallenge. The patient cohort had a mean age of 59.3 ± 10.0 years, with 55% of participants being women. Patients predominantly reported rest angina (55%), followed by mixed rest and exertional angina (32.5%), with the remaining presenting with exertional angina (12.5%). The patient cohort was diverse in ethnicity, with the majority being Caucasian (67.5%). Clinical, laboratory, and medication data are detailed in Table 1.

**Table 1:** Patient characteristics.

Characteristics	N = 40
Age, y	59.3 ± 10.0
Female sex	22 (55%)
Body mass index, kg/m^2^	26.2 ± 3.4
Ethnicity	
Caucasian	27 (67.5%)
Asian	4 (10.0%)
Southeast Asian	4 (10.0%)
Middle Eastern	5 (12.5%)
Smoking status	
Never	27 (67.5%)
Former	9 (22.5%)
Current	4 (10.0%)
Hypertension	18 (45.0%)
Hypercholesterolemia	25 (62.5%)
Diabetes	9 (22.5%)
Overweight	19 (47.5%)
Family history of CAD	12 (30.0%)
Peripheral vascular disease	1 (2.5%)
CAD	3 (7.5%)
Previous invasive coronary angiogram	18 (45.0%)
Cerebrovascular accident	1 (2.5%)
Obstructive sleep apnea	3 (7.5%)
Migraines	10 (25.0%)
Chronic pain syndromes	8 (20.0%)
Preventative therapy	
Aspirin	28 (70.0%)
ACE-I/ARB	16 (40.0%)
Statin	23 (57.5%)
Angina therapy	
Beta-blocker	18 (45.0%)
Calcium channel blockers (withheld prior to testing)	10 (25.0%)
Nitrates (withheld prior to testing)	10 (25.0%)
Nicorandil (withheld prior to testing)	4 (10.0%)
Laboratory	
Total cholesterol	3.70 ± 0.62
Low-density lipoprotein	1.85 ± 0.71
High-density lipoprotein	1.11 ± 0.41
Triglycerides	1.55 ± 0.65
HbA1c	5.55 ± 0.71
CCS angina class	
1	10 (25.0%)
2	16 (40.0%)
3	11 (27.5%)
4	2 (5.0%)
Angina pattern	
Predominately rest	22 (55.0%)
Predominately exertion	5 (12.5%)
Mixed	13 (32.5%)
Circadian pattern	
Predominately daytime (7 AM to 7 PM)	12 (30.0%)
Predominately nocturnal (7 PM to 7 AM)	14 (35.0%)
Throughout the day	14 (35.0%)
Stress echocardiography	
Normal	22 (59.5%)
Inconclusive	1 (2.70%)
Abnormal	14 (37.8%)
Radionuclide myocardial perfusion	
Negative or inconclusive	2 (66.7%)
Abnormal	1 (33.3%)

Values are mean ± SD or n (%).

ACE-I, angiotensin-converting enzyme inhibitor; ARB, angiotensin receptor blocker; CAD, coronary artery disease; CCS, Canadian Cardiovascular Society.

### Procedural Characteristics

All coronary angiograms were initially attempted via radial arterial access; however, 15% of cases required conversion to femoral access due to procedural challenges. In all patients, NTG was administered via initial radial access prior to ACh rechallenge.

More than half of the patients (55%) demonstrated angiographically normal coronary arteries. The most prevalent coronary circulation pattern was right-dominant (75%), followed by left-dominant (15%) and codominant (10%). There were no serious adverse events. During ACh provocation testing, backup pacing was required in 6 patients (15%) and 21 patients (52.5%) during LCA and RCA assessment, respectively. Transient atrial fibrillation occurred in 4 patients (10%), with all cases resolving spontaneously on the day of the procedure. No additional sedation was required during ACh testing. Procedural characteristics are detailed in Supplemental Table S2.

### ACh provocation testing

In the study cohort, a diffuse spasm pattern was identified in 25 patients (62.5%), whereas the remaining 15 patients (37.5%) exhibited focal spasm (Table 2). Epicardial CAS was observed in the left anterior descending artery in 90% (36/40) of patients, followed by the left circumflex artery in 27.5% (11/40) and RCA in 25% (10/40) of patients. Multivessel spasm was present in 15 (37.5%) patients. When evaluating the dose-response relationship in the LCA (n = 37), CAS occurred in 10.8% of patients at 20 μg, 27% at 50 μg, 48.6% at 100 μg, and 13.6% at 200 μg. For the RCA (n = 10), CAS occurred in 40% of patients at 20 μg, 40% at 50 μg, and 20% at 80 μg.

**Table 2:** Summary of invasive ACh provocation testing.

	N = 40
ACh testing	
Epicardial spasm	40 (100%)
Diffuse spasm	25 (62.5%)
Focal spasm	15 (37.5%)
Location of spasm	
Left anterior descending artery	36 (90.0%)
Left circumflex artery	11 (27.5%)
Right coronary artery	10 (25.0%)
Multivessel	15 (37.5%)
Dose required to provoke spasm	
Left coronary artery	n = 37
20 μg	4 (10.8%)
50 μg	10 (27.0%)
100 μg	18 (48.6%)
200 μg	5 (13.6%)
Right coronary artery	n = 10
20 μg	4 (40.0%)
50 μg	4 (40.0%)
80 μg	2 (20.0%)
ACh rechallenge	
5 Minutes	
Epicardial spasm	12 (30.0%)
Microvascular spasm	2 (5.00%)
No spasm	26 (65.0%)
10 Minutes^a^	
Epicardial spasm	10 (25.0%)
Microvascular spasm	4 (10.0%)
No spasm	14 (35.0%)

Values are n (%).

ACh, acetylcholine.

a Only patients (n = 28) who did not demonstrate epicardial coronary artery spasm at the 5-minute ACh rechallenge proceeded to the 10-minute ACh rechallenge.

### ACh rechallenge

All patients underwent ACh rechallenge 5 minutes after intraradial NTG administration. Epicardial spasm was reinduced in 12 patients (30%), microvascular spasm was observed in 2 patients (5%), and 26 patients (65%) showed no evidence of CAS. Subsequent ACh rechallenge at 10 minutes was performed in the latter 28 patients (70%) who did not exhibit epicardial spasm during the initial rechallenge. During subsequent testing, epicardial spasm was reinduced in 10 patients (25%), microvascular spasm was observed in 4 patients (10%), and 14 patients (35%) showed no evidence of spasm (Central Illustration).

**Central Illustration:**
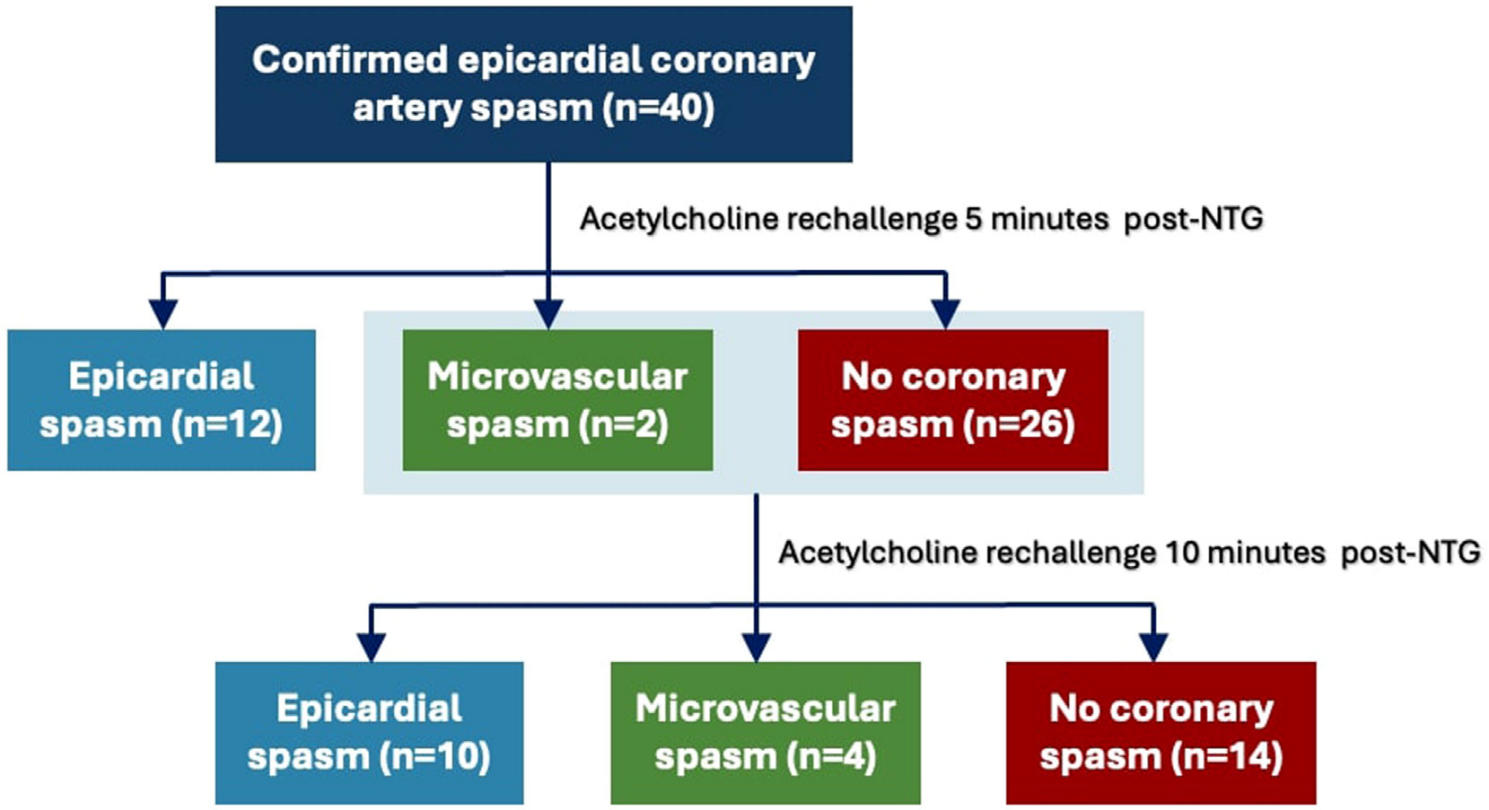
**Results of acetylcholine rechallenge.** At 5 minutes, nitroglycerin (NTG) was effective in preventing epicardial spasms in 28 of 40 (70%) patients. At 10 minutes, nitroglycerin was effective in preventing epicardial spasms in 18 of 40 (45%) patients.

Supplemental Figure S1 demonstrates the time-dependent relationship between ACh and the %MLD change. At 5 minutes post-NTG administration, the average %MLD reduction was 47% (±0.30%). This reduction increased to 64% (±0.24%) at the 10-minute mark, indicating a progressive vasoconstrictive response over time. Quantitative coronary angiography (QCA) results during the ACh rechallenge are summarized in Supplemental Table S3.

## Discussion

This study is the first to systematically evaluate the impact of intravascular NTG administration on downstream ACh provocation testing in patients with ANOCA. The major finding of our study is that the sensitivity of ACh provocation testing is reduced to 30% and 55% at 5 minutes and 10 minutes post-NTG administration, respectively. These results suggest that NTG administration reduces the diagnostic efficacy of ACh provocation testing for CAS.

Patients with ANOCA often experience severe and recurrent symptoms, resulting in significantly impaired quality of life, frequent hospitalizations for chest pain, and repeated investigations.^1^^,^^17^^,^^18^ Accurate identification of ANOCA endotypes through CFT is essential for tailoring optimal pharmacological therapy in this patient population.^4^ Although the European Society of Cardiology and the American College of Cardiology/American Heart Association guidelines recommend CFT,^5^^,^^19^ there remains global variability in its implementation, particularly regarding the exact sequence of ACh provocation and CMD testing.

Some institutions advocate for assessing the coronary microcirculation prior to ACh provocation due to its potential confounding influence on subsequent physiological measurements. Previous studies have reported that resting mean transit time and baseline resistance index are elevated in patients with a positive ACh provocation test compared with those with a negative response.^11^^,^^20^ These findings have also been corroborated by studies using transthoracic Doppler echocardiography and intracoronary dual-sensor guide wires^21^^,^^22^ (measuring Doppler velocity and pressure).

Conversely, although NTG administration is crucial for achieving maximal vasodilation during CMD assessment, it may inadvertently lead to the underdiagnosis of CAS.^12^^,^^13^ NTG acts primarily on larger conduit coronary arteries through the nitric oxide pathway, with its vasodilatory effects diminishing as vessel size decreases.^23^ Nitrates have been shown to exert vasodilatory effects on epicardial coronary arteries for approximately 10 to 15 minutes.^12^ Seitz et al^24^ demonstrated that after significant epicardial spasm was induced by a dose of ACh provocation, readministration of ACh at the same dose 3 minutes post-NTG failed to reinduce focal epicardial vasospasm and was highly effective in preventing diffuse epicardial spasm. This finding implies a 3-minute interval is insufficient for accurate CAS diagnosis following NTG administration. We designed our study to assess the effect of ACh administration after 5 and 10 minutes of intraradial NTG, which represents the approximate time it takes to perform the CMD assessment in an experienced CFT lab. Our results demonstrated that intracoronary NTG prevented reproduction of epicardial CAS in 70% and 45% of patients at the 5- and 10-minute time-points, respectively.

**Figure 2:**
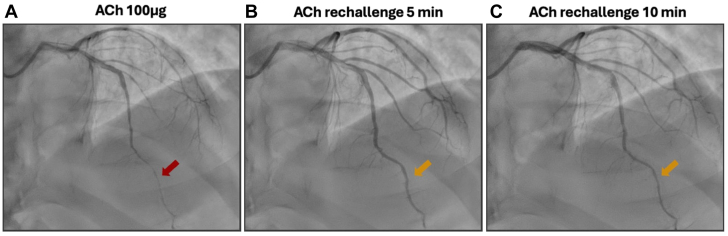
**Intracoronary acetylcholine rechallenge at 5 and 10 minute intervals.(A)** Diffuse epicardial spasm of the left anterior descending artery (red arrow) provoked by intracoronary acetylcholine (Ach) 100 μg. ACh rechallenge at 5 minutes **(B)** and 10 minutes **(C)** demonstrated a progressive vasoconstrictive response (gold arrows) without reaching >90% diameter stenosis threshold for epicardial spasm.

Furthermore, our study revealed a time-dependent reduction in minimum lumen diameter (MLD) within the epicardial vessels following intravascular NTG administration. At 5 minutes post-NTG, the mean MLD reduction was 47% (±0.30%), increasing to 64% (±0.24%) at 10 minutes, demonstrating a heightened vasoconstrictive response with delayed reexposure to ACh (Figure 2). This finding suggests that a longer interval between NTG administration and subsequent ACh provocation should at least be considered, or alternatively, that NTG should be avoided prior to ACh testing.

Acetylcholine rechallenge also identified concomitant microvascular spasm in 15% (6/40) of patients, a finding consistent with previous research.^24^ As noted by Sellke et al,^25^ NTG demonstrates reduced efficacy in preventing spasm within the coronary microcirculation compared with its more potent effects on the epicardial circulation. The limited effect in smaller coronary microvessels may be attributed to diminished biotransformation of NTG in these vessels. This observation highlights the heterogeneity and complexity of vasomotor disorders in patients with ANOCA. Recognizing additional diagnoses, such as microvascular spasm, is crucial for clinicians, as it has significant treatment implications.^26^^,^^27^

Notably, global variability in ACh administration protocols may affect diagnostic outcomes. Slow ACh infusions (2-3 minutes) are commonly used to assess endothelial function, whereas rapid injections (20-30 seconds), as employed in this study, are designed to diagnose clinically relevant CAS.^28^ Rapid administration may provoke immediate endothelial and smooth muscle activation, leading to the release of vasoconstrictive mediators, whereas slower infusion may induce a more gradual response, potentially leading to an underestimation of CAS.^7^

### Limitations

First, our sample size is relatively small, underscoring the need for validation of our findings in a larger cohort. Second, the timing of the ACh rechallenge was limited to 5 and 10 minutes post-NTG administration. Although this protocol provided valuable data on the time-dependent response to ACh, it did not explore longer intervals, which may have offered further insights. Third, we cannot exclude tachyphylaxis as the mechanism of failed CAS provocation during the ACh rechallenge. Furthermore, we used the same dose of ACh that initially provoked epicardial CAS during the ACh rechallenge. Employing higher doses during ACh rechallenge may have potentially increased the detection rate of epicardial CAS. Lastly, our study did not assess the clinical implications of vasomotor responses, particularly whether patients with reinducible spasm, post-NTG administration, are less responsive to conventional therapy.

## Conclusion

In patients with ANOCA, NTG administration can diminish the effectiveness of ACh provocation testing in diagnosing CAS. Our findings suggest clinicians should avoid NTG administration before performing ACh provocation testing or delay ACh testing significantly longer than 10 minutes after NTG administration. Future studies should aim to define the appropriate sequential order for CFT in patients with ANOCA.
