# Supplementary material for: Impact of Nitroglycerin Administration on Acetylcholine Provocation Testing in Angina With Nonobstructive Coronary Arteries
Source: J Soc Cardiovasc Angiogr Interv. 2025 Jul 23;4(8):103668. doi: 10.1016/j.jscai.2025.103668 (PMC12462065; doi:10.1016/j.jscai.2025.103668)
Supplement: Supplementary Figure 1 and Tables 1-3 [file mmc1.docx]

**Supplemental Materials**

**Supplemental Figure 1:** Time-response relationship between intracoronary acetylcholine and % minimal lumen diameter change

MLD = minimal lumen diameter, NTG = nitroglycerin

**
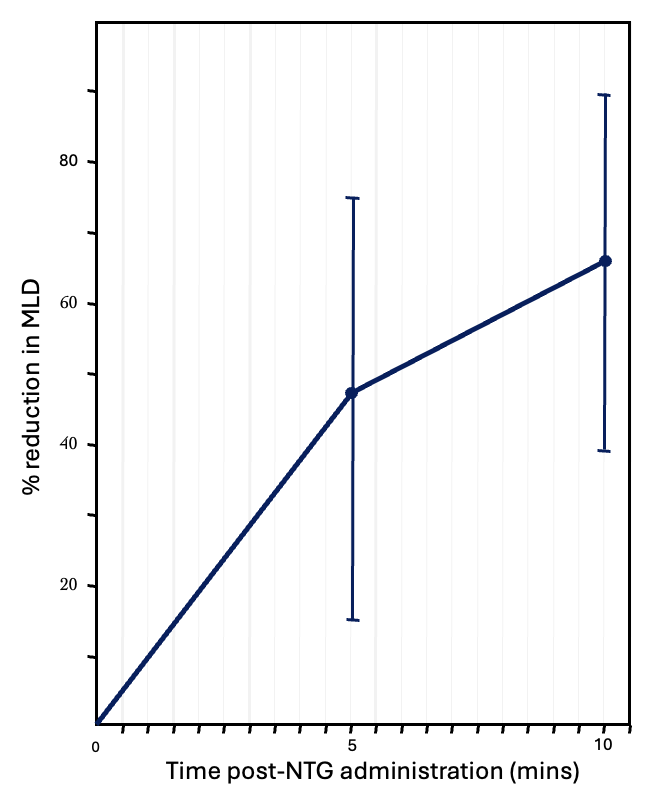
**

**Supplemental Table 1: Quantitative coronary angiography following ACh rechallenge**

| **Patient** | **Rechallenge 5min QCA (%)** | **Rechallenge 10min QCA (%)** |
| --- | --- | --- |
| Patient 01 | 17 | 46 |
| Patient 02 | 56 | 91 |
| Patient 03 | 48 | 93 |
| Patient 04 | 29 | 50 |
| Patient 05 | 28 | 59 |
| Patient 06 | 91 | NA |
| Patient 07 | 46 | 91 |
| Patient 08 | 27 | 92 |
| Patient 09 | 38 | 93 |
| Patient 10 | 38 | 91 |
| Patient 11 | 25 | 53 |
| Patient 12 | 48 | 92 |
| Patient 13 | 16 | 51 |
| Patient 14 | 91 | NA |
| Patient 15 | 31 | 91 |
| Patient 16 | 50 | 66 |
| Patient 17 | 40 | 91 |
| Patient 18 | 27 | 47 |
| Patient 19 | 92 | NA |
| Patient 20 | 94 | NA |
| Patient 21 | 39 | 59 |
| Patient 22 | 30 | 57 |
| Patient 23 | 14 | 39 |
| Patient 24 | 19 | 45 |
| Patient 25 | 93 | NA |
| Patient 26 | 12 | 31 |
| Patient 27 | 10 | 26 |
| Patient 28 | 34 | 49 |
| Patient 29 | 23 | 49 |
| Patient 30 | 91 | NA |
| Patient 31 | 35 | 93 |
| Patient 32 | 13 | 43 |
| Patient 33 | 94 | NA |
| Patient 34 | 93 | NA |
| Patient 35 | 92 | NA |
| Patient 36 | 13 | 31 |
| Patient 37 | 91 | NA |
| Patient 38 | 92 | NA |
| Patient 39 | 20 | 42 |
| Patient 40 | 92 | NA |

QCA = quantitative coronary angiography

**Supplemental Table 2: Procedural Characteristics**

| **Characteristics** | **All patients (n=40)** |
| --- | --- |
| **Final Access Site** | |
| Radial | 34 (85%) |
| Femoral | 6 (15%) |
| **Coronary dominance** | |
| Right-dominant | 30 (75%) |
| Left-dominant | 6 (15%) |
| Co-dominant | 4 (10%) |
| **Complication** | |
| Atrial Fibrillation | 4 (10%) |
| **Activation of TTP** | |
| LCA | 6 (15%) |
| RCA | 21 (52.5%) |

LCA = left coronary artery, RCA = right coronary artery, TTP = temporary transvenous pacemaker

**Supplemental Table 3: Procedural Characteristics**

| **Patient** | **RFR** | **FFR** | **IMR** | **CFR** |
| --- | --- | --- | --- | --- |
| Patient 01 | 0.88 | 0.84 | 8 | 2.8 |
| Patient 02 | 0.93 | 0.97 | 6 | 3.2 |
| Patient 03 | 0.92 | 0.89 | 26 | 2.1 |
| Patient 04 | 0.89 | 0.86 | 21 | 1.9 |
| Patient 05 | 0.9 | 0.86 | 13 | 4.3 |
| Patient 06 | 0.92 | 0.84 | 10 | 4.5 |
| Patient 07 | 0.95 | 0.94 | 7 | 2.7 |
| Patient 08 | 0.94 | 0.92 | 26 | 2.2 |
| Patient 09 | 0.9 | 0.93 | 34 | 3.1 |
| Patient 10 | 0.93 | 0.88 | 27 | 2.4 |
| Patient 11 | 0.9 | 0.89 | 17 | 3.1 |
| Patient 12 | 0.92 | 0.82 | 12 | 7.1 |
| Patient 13 | 0.95 | 0.94 | 7 | 5.4 |
| Patient 14 | 0.92 | 0.91 | 23 | 1.6 |
| Patient 15 | 0.9 | 0.85 | 15 | 4.5 |
| Patient 16 | 0.94 | 0.91 | 18 | 5.1 |
| Patient 17 | 0.93 | 0.91 | 18 | 3.3 |
| Patient 18 | 0.87 | 0.82 | 16 | 2.8 |
| Patient 19 | 0.89 | 0.85 | 29 | 2 |
| Patient 20 | 0.94 | 0.95 | 19 | 6.1 |
| Patient 21 | 0.93 | 0.86 | 21 | 1.9 |
| Patient 22 | 0.92 | 0.85 | 20 | 2.5 |
| Patient 23 | 0.99 | 0.97 | 26 | 2 |
| Patient 24 | 0.91 | 0.89 | 38 | 1.7 |
| Patient 25 | 0.96 | 0.93 | 39 | 2.9 |
| Patient 26 | 0.95 | 0.85 | 32 | 1.9 |
| Patient 27 | 0.94 | 0.99 | 36 | 8.7 |
| Patient 28 | 0.92 | 0.87 | 20 | 3.4 |
| Patient 29 | 0.91 | 0.87 | 31 | 1.8 |
| Patient 30 | 0.93 | 0.91 | 28 | 2.8 |
| Patient 31 | 0.93 | 0.9 | 31 | 2.3 |
| Patient 32 | 0.94 | 0.93 | 41 | 2.6 |
| Patient 33 | 0.9 | 0.89 | 55 | 1.5 |
| Patient 34 | 0.98 | 0.96 | 31 | 3 |
| Patient 35 | 0.93 | 0.9 | 14 | 1.5 |
| Patient 36 | 0.92 | 0.9 | 12 | 6.5 |
| Patient 37 | 0.88 | 0.84 | 8 | 2.8 |
| Patient 38 | 0.93 | 0.97 | 6 | 3.2 |
| Patient 39 | 0.92 | 0.89 | 26 | 2.1 |
| Patient 40 | 0.89 | 0.86 | 21 | 1.9 |

LCA = left coronary artery, RCA = right coronary artery, TTP = temporary transvenous pacemaker
